# Grape-Derived Polyphenols Prevent Doxorubicin-Induced Blunted EDH-Mediated Relaxations in the Rat Mesenteric Artery: Role of ROS and Angiotensin II

**DOI:** 10.1155/2013/516017

**Published:** 2013-08-28

**Authors:** Noureddine Idris-Khodja, Paola Di Marco, Mona Farhat, Bernard Geny, Valérie B. Schini-Kerth

**Affiliations:** ^1^UMR CNRS 7213, Laboratoire de Biophotonique et Pharmacologie, Faculté de Pharmacie, Université de Strasbourg, 74 Route du Rhin, BP 60024, 67401 Illkirch, France; ^2^Institut de Physiologie, Faculté de Médecine, EA 3072, 11 Rue Humann, 67000 Strasbourg, France; ^3^Service de Physiologie et d'Explorations Fonctionnelles, Pôle de Pathologie Thoracique, NHC-1 Place de l'Hôpital, 67091 Strasbourg, France

## Abstract

This study determined whether doxorubicin, an anticancer agent, impairs endothelium-dependent relaxations mediated by nitric oxide (NO) and endothelium-derived hyperpolarization (EDH) in the mesenteric artery and, if so, the mechanism underlying the protective effect of red wine polyphenols (RWPs), a rich natural source of antioxidants. Male Wistar rats were assigned into 4 groups: control, RWPs, doxorubicin, and doxorubicin + RWPs. Vascular reactivity was assessed in organ chambers; the vascular formation of reactive oxygen species (ROS) using dihydroethidine and the expression levels of small and intermediate conductance calcium-activated potassium channels (SK_Ca_, IK_Ca_) and connexin 40 (Cx40), which are involved in EDH-type relaxations, endothelial NO synthase (eNOS), angiotensin II, and AT1 receptors by immunofluorescence. The doxorubicin treatment impaired EDH-mediated relaxations, whereas those mediated by NO were minimally affected. This effect was associated with reduced expression levels of SK_Ca_, IK_Ca_, and Cx40, increased expression levels of eNOS, angiotensin II, and AT1 receptors, and formation of ROS in mesenteric arteries. RWPs prevented both the doxorubicin-induced blunted EDH-type relaxations and the increased vascular oxidative stress, and they improved the expression levels of target proteins. These findings suggest that polyphenol-rich natural products might be of interest in the management of doxorubicin-induced vascular injury possibly by improving the vascular angiotensin system.

## 1. Introduction

Doxorubicin is a highly effective anticancer agent with a broad spectrum of activity in human cancers, which is often used for the treatment of solid tumors and malignant hematological diseases [1]. However, a major limitation of doxorubicin treatment is its dose-dependent cardiotoxicity [2]. Doxorubicin has been shown to induce oxidative stress which can lead to dilated cardiomyopathy with the subsequent development of left ventricular dysfunction and congestive heart failure [3]. Besides affecting the heart, doxorubicin appears also to impair the vascular function by inducing an endothelial dysfunction [4, 5]. In healthy blood vessels, the endothelium plays a major role in vascular homeostasis mostly by generating the formation of nitric oxide (NO), a potent vasodilator synthesized by endothelial NO synthase (eNOS) [6–9], and by inducing endothelium-dependent hyperpolarization- (EDH-) mediated relaxations [10]. The EDH component of endothelium-dependent relaxations increases as the size of the artery decreases [11] and involves the activation of endothelial SK_Ca_ and IK_Ca_ channels (small and intermediate conductance Ca^2+^-activated K^+^ channels, resp.) inducing hyperpolarization of the endothelium. In some types of blood vessels, endothelial hyperpolarization is transmitted to the underlying vascular smooth muscle cells via myoendothelial gap junctions with the subsequent relaxation [12]. In others, the K^+^ that effluxes through SK_Ca_ activates myocytes and endothelial Ba^2+^-sensitive K_IR_ channels leading to myocyte hyperpolarization [12]. K^+^ effluxing through IK_Ca_ can also activate ouabain-sensitive Na^+^/K^+^-ATPases generating further myocyte hyperpolarization [12]. Gap junctions are formed by the docking of two apposing connexons, which are composed of six connexins (Cx) either from one or more of the following three connexin proteins: Cx37, Cx40, and Cx43 [13]. Previous studies have shown that doxorubicin impairs NO-mediated relaxations in the rabbit [14] and rat aortae [4] and decreases brachial artery flow-mediated vasodilation in adult [14] and pediatric cancer patients [15]. Other studies have indicated that doxorubicin-induced cardiotoxicity is due, at least in part, to ROS formation [3]. Furthermore, the activation of the angiotensin system has been suggested to play a key role in the development of the doxorubicin-induced cardiotoxicity since an improved heart function was observed in mice treated with either an AT1 receptor antagonist [16] or an angiotensin-converting enzyme inhibitor [17] and was also observed in AT1 receptor knockout mice [16].

Polyphenols are natural antioxidants present in high levels in tea, chocolate, fruits, and vegetables, and their intake has been associated with vascular protective effects [18, 19]. Beside their well-known antioxidant properties, polyphenols such as those from red wine (RWPs) protect also the endothelial function by stimulating endothelium-dependent NO- and EDH-mediated relaxations and by preventing vascular oxidative stress [20]. Indeed, RWPs prevented endothelial dysfunction and oxidative stress in the aortae of angiotensin II-induced hypertension [21], deoxycorticosterone acetate salt-induced hypertension [22], and spontaneously hypertensive rats [23], and also in the mesenteric arteries of old rats [24]. RWPs have also been shown to normalize the excessive vascular expression levels of both angiotensin II and AT1 receptor in old rats [24]. Therefore, the aim of the present study was to determine whether chronic treatment of rats with doxorubicin alters the NO- and EDH-mediated relaxations in the mesenteric artery, and, if so, to evaluate the potential protective effect of red wine polyphenols and to clarify the underlying mechanism.

## 2. Methods

### 2.1. *In Vivo* Treatment of Rats

This study conforms to the Guide of Care and the Use of Laboratory Animals published by the US National Institutes of Health (NIH Publication no. 85-23, revised in 1996), and the protocol was approved by the local ethical committee. Food and water were given *ad libitum* in a controlled environment (room temperature 21-22°C and room humidity 50% ± 5%). Forty male Wistar rats (12-week old) were used. Rats were randomly assigned into 4 groups of 10 rats: Group 1 (vehicle) received intraperitoneal (IP) injections of the doxorubicin vehicle (dimethyl sulfoxide, DMSO) and the RWPs-solvent (ethanol, 3% v/v) in the drinking water starting at the age of 12 weeks; Group 2 (RWPs) received IP injections of the doxorubicin vehicle (DMSO) and RWPs (75 mg/kg/day) in the drinking water starting at the age of 12 weeks; Group 3 (doxorubicin) received IP injections of doxorubicin (2.5 mg/kg/week) for 3 subsequent weeks starting at the age of 12 weeks followed again by IP injections (2.5 mg/kg/week) for 3 subsequent weeks starting at the age of 28 weeks, with the RWPs-solvent (ethanol, 3% v/v) in the drinking water starting at the age of 12 weeks; Group 4 (doxorubicin + RWPs) was treated with doxorubicin and RWPs. At the age of 34 weeks, the rats were anaesthetized with pentobarbital (50 mg/kg, IP). After excision, the mesenteric artery was placed in the Krebs bicarbonate solution for the subsequent determination of the vascular reactivity using organ chambers, the expression levels of target proteins by immunofluorescence, and the vascular oxidative stress using the redox-sensitive probe dihydroethidine (DHE).

### 2.2. Vascular Reactivity Studies

The main superior mesenteric artery was cleaned of connective tissue and cut into rings (2-3 mm in length). In some preparations, the endothelium was removed by rubbing the intimal surface of rings with a pair of forceps. Rings were suspended in organ baths containing oxygenated (95% O_2_ and 5% CO_2_) Krebs bicarbonate solution (mM: NaCl 119, KCl 4.7, KH_2_PO_4_ 1.18, MgSO_4_ 1.18, CaCl_2_ 1.25, NaHCO_3_ 25, and d-glucose 11; pH 7.4 and 37°C) for the determination of changes in isometric tension. Rings were stretched step by step until an optimal resting tension of 1 g was reached and then allowed to equilibrate for at least 60 min. After the equilibration period, rings were exposed to the high K^+^-containing Krebs bicarbonate solution (80 mM) until reproducible contractile responses were obtained. After washing with the Krebs bicarbonate solution, rings were precontracted with phenylephrine (PE, 1 *μ*M) to about 80% of the maximal contraction induced by the high K^+^-containing Krebs bicarbonate solution before addition of acetylcholine (ACh, 1 *μ*M) to check the presence of a functional endothelium. After washout and a further 30 min equilibration period, rings were again contracted with PE before the application of increasing concentrations of ACh (0.1 nM to 10 *μ*M), sodium nitroprusside (an exogenous NO donor, 0.1 nM to 1 *μ*M), or levcromakalim (an ATP-sensitive K^+^ channel opener, 0.1 nM to 10 *μ*M) to construct concentration-response curves. Sodium nitroprusside- and levcromakalim-induced relaxations were examined in endothelium-denuded rings of mesenteric artery to investigate the reactivity of the vascular smooth muscle. In some experiments, rings were exposed to an inhibitor for 30 min before the contraction with PE. The NO component of relaxation was determined in the presence of indomethacin (10 *μ*M) and in the presence of charybdotoxin (100 nM) plus apamin (100 nM) to inhibit the participation of prostanoids and EDH, respectively. The EDH component of relaxation was determined in the presence of indomethacin (10 *μ*M) and in the presence of N^*ω*^-nitro-L-arginine (L-NA, 300 *μ*M) to inhibit the formation of prostanoids and NO, respectively. Relaxations were expressed as a percentage of the contraction induced by PE.

### 2.3. Immunofluorescence Studies

Short segments of main mesenteric arteries were removed, embedded in OCT compound (Tissue-Tek, Sakura Finetek), and snap-frozen in liquid nitrogen. Frozen arteries were cryosectioned at 14 *μ*m. Sections were air dried for 15 min and stored at −80°C until used. Sections were first fixed with paraformaldehyde at 4%, washed, and treated with 10% milk or 5% goat serum in PBS containing 0.1% Triton X100 for 1 h at room temperature to block nonspecific binding. Sections were then incubated overnight at 4°C with an antibody directed against either calcium-dependent potassium channels (1/200), connexin 40 (1/200), eNOS (1/200), angiotensin II (1/500), or AT1 receptors (1/400). Sections were then washed with PBS, incubated with the secondary antibody (Alexa 637-conjugated goat anti-mouse IgG or Alexa 637-conjugated goat anti-rabbit, 1/400) diluted in the same buffer for 2 h at room temperature in the dark, and washed before being mounted in Vectashield (mounting medium for fluorescence, Vector Laboratories, Inc., Burlingame, CA, USA) and coverslipped. For negative controls, primary antibodies were omitted. The sections were observed using a confocal laser-scanning microscope (Leica SP2 UV DM IRBE). Quantification of fluorescent levels was performed using Image J 1,42q software (National Institutes of Health, USA).

### 2.4. Determination of Vascular Oxidative Stress

The redox-sensitive fluorescent dye DHE was used to evaluate the *in situ* formation of ROS. Mesenteric artery rings (3 to 4 mm in length) were embedded in OCT compound and frozen in a nitrogen bath for cryostat sections. DHE (2.5 *μ*M) was applied onto 25 *μ*m unfixed cryosections of mesenteric arteries for 30 min at 37°C in a light-protected humidified chamber to determine the *in situ* formation of ROS. To determine the nature and source of ROS, rings were incubated with several inhibitors including MnTMPyP (membrane-permeant superoxide dismutase mimetic, 100 *μ*M), polyethylene glycol-catalase (membrane-permeant catalase, 500 UI/mL), L-NA (NO synthase inhibitor, 300 *μ*M), apocynin (NADPH oxidase inhibitor and antioxidant, 300 *μ*M), and inhibitors of the mitochondrial respiration chain (myxothiazol, 0.5 *μ*M + rotenone, 1 *μ*M + KCN, 1 *μ*M) for 30 min at 37°C before adding DHE. Sections were then washed three times, mounted in Vectashield, and coverslipped. Images were obtained with a Leica SP2 UV DM IRBE laser-scanning confocal microscope. Quantification of staining levels was performed using Image J 1,42q software.

### 2.5. Materials

Antibodies were purchased as indicated: anti-mouse Cx40 polyclonal antibody (Chemicon, Temecula, CA, USA), anti-KCa3.1 (intermediate conductance Ca^2+^-activated K^+^ channel 4, IKCa), and anti-KCa2.3 (small conductance Ca^2+^-activated K^+^ channel 3, SKCa) (Alomone Labs, Jerusalem, Israel); mouse anti-eNOS (BD Transduction Laboratories, San Jose, CA, USA); rabbit anti-angiotensin II (Peninsula Laboratories, San Carlos, CA), rabbit anti-AT1 receptor (Santa Cruz Biotechnology); and Alexa fluor-488 or 637 labeled goat anti-rabbit IgG (Invitrogen, Molecular Probes). All chemicals were obtained from Sigma-Aldrich except for apamin and charybdotoxin, which were purchased from Latoxan (Valence, France).

### 2.6. Preparation of Red Wine Polyphenols (RWPs)

RWPs dry powder, obtained from the French red wine (Corbières AOC., France), was provided by Dr. M. Moutounet (Institut National de la Recherche Agronomique, Montpellier, France) and analyzed by Prof. P.-L. Teissedre (Université de Bordeaux, France). One liter of red wine produced 2.9 g of RWPs, which contained 471 mg/g total phenolic compounds expressed as gallic acid equivalents. The extract contained 8.6 mg/g catechin, 8.7 mg/g epicatechin, dimers (B1, 6.9 mg/g; B2, 8.0 mg/g; B3, 20.7 mg/g; B4, 0.7 mg/g), anthocyanins (malvidin-3-glucoside, 11.7 mg/g; peonidin-3-glucoside, 0.66 mg/g; cyanidin-3-glucoside, 0.06 mg/g), and phenolic acids (gallic acid, 5.0 mg/g; caffeic acid, 2.5 mg/g; caftaric acid, 12.5 mg/g).

### 2.7. Statistical Analysis

Data are presented as mean ± SEM of *n* different experiments. Mean values were compared using ANOVA followed by the post hoc Bonferroni test to identify significant differences between treatments, using GraphPad Prism (version 5 for Microsoft Windows, GraphPad software, Inc., San Diego, CA, USA). The difference was considered to be significant when the *P* value was less than 0.05.

## 3. Results

### 3.1. RWPs Treatment Prevents the Doxorubicin-Induced Blunted EDH-Mediated Relaxations in the Rat Mesenteric Artery

**Figure 1:**
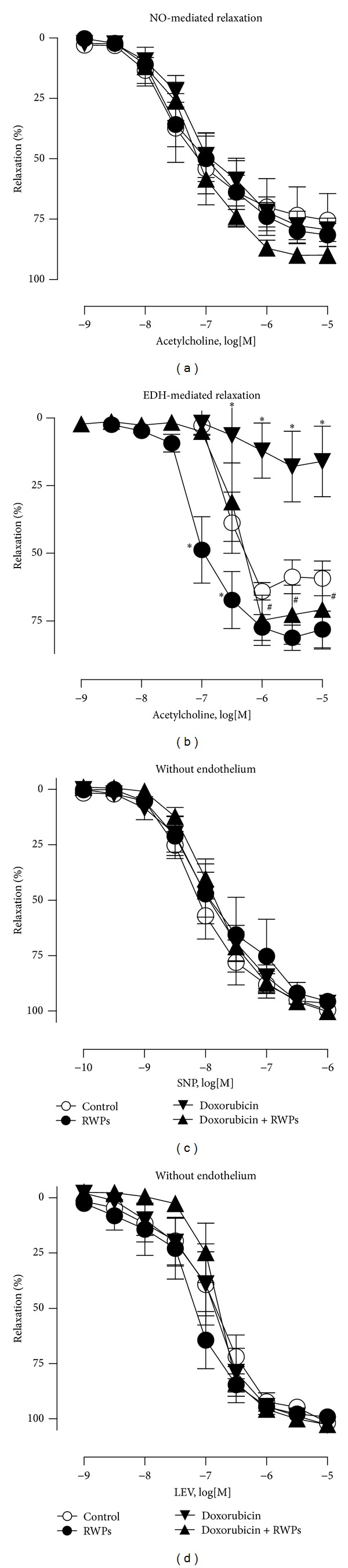
RWPs treatment prevents the doxorubicin-induced blunted EDH-mediated relaxations in the rat mesenteric artery. Twelve-week-old male Wistar rats were assigned into 4 groups: the control group receiving the doxorubicin vehicle (DMSO, IP) and the RWPs-solvent (ethanol 3% V/V, *per os*); the RWPs group receiving the doxorubicin vehicle and RWPs (75 mg/kg/day, *per os*); the doxo group receiving doxorubicin (2.5 mg/kg/week, IP) for 3 subsequent weeks starting at the age of 12 weeks followed again by injections (2.5 mg/kg/week) for 3 subsequent weeks starting at the age of 28 weeks, with the RWPs solvent *per os*; the doxorubicin + RWPs group receiving doxorubicin and RWPs. Mesenteric artery rings with endothelium from the indicated groups of rats were contracted with phenylephrine in the presence of indomethacin (10 *μ*M) to inhibit the formation of prostanoids, in the presence of (a) charybdotoxin (100 nM) plus apamin (100 nM) to inhibit the participation of EDH, in the presence of (b) N^*ω*^-nitro-L-arginine (L-NA, 300 *μ*M) to rule out the formation of NO before a concentration-relaxation curve to ACh was constructed. Concentration-relaxation curves to sodium nitroprusside (SNP, (c)) and levcromakalim (LEV, (d)) in mesenteric artery rings without endothelium are demonstrated. Results are shown as mean ± SEM of 5 to 6 different rats. *P < 0.05 indicates a significant difference versus control rats, and ^#^ *P* < 0.05 versus doxorubicin-treated rats.

In mesenteric artery rings with endothelium, the doxorubicin treatment did not significantly affect the ACh-induced NO-mediated relaxations as assessed in the presence of indomethacin and in the presence of charybdotoxin plus apamin to prevent the formation of vasoactive prostanoids and EDH, respectively (Figure 1(a)). In contrast, the ACh-induced EDH-mediated relaxations as assessed in the presence of indomethacin and L-NA to prevent the formation of vasoactive prostanoids and NO, respectively, were markedly reduced in the doxorubicin-treated rats compared with the control rats (Figure 1(b)). Intake of RWPs (75 mg/kg/day) in the drinking water prevented the inhibitory effect of the doxorubicin treatment on EDH-mediated relaxations, and it also slightly but significantly increased EDH-mediated relaxations in control rats, whereas NO-mediated relaxations were not affected (Figures 1(a) and 1(b)). Neither the doxorubicin treatment nor the RWPs treatment affected relaxations to sodium nitroprusside or levcromakalim in mesenteric artery rings without endothelium (Figures 1(c) and 1(d)).

### 3.2. RWPs Treatment Prevents the Doxorubicin-Induced Impaired Expression Levels of SK_Ca_, IK_Ca_, and Cx40 in the Mesenteric Artery

**Figure 2:**
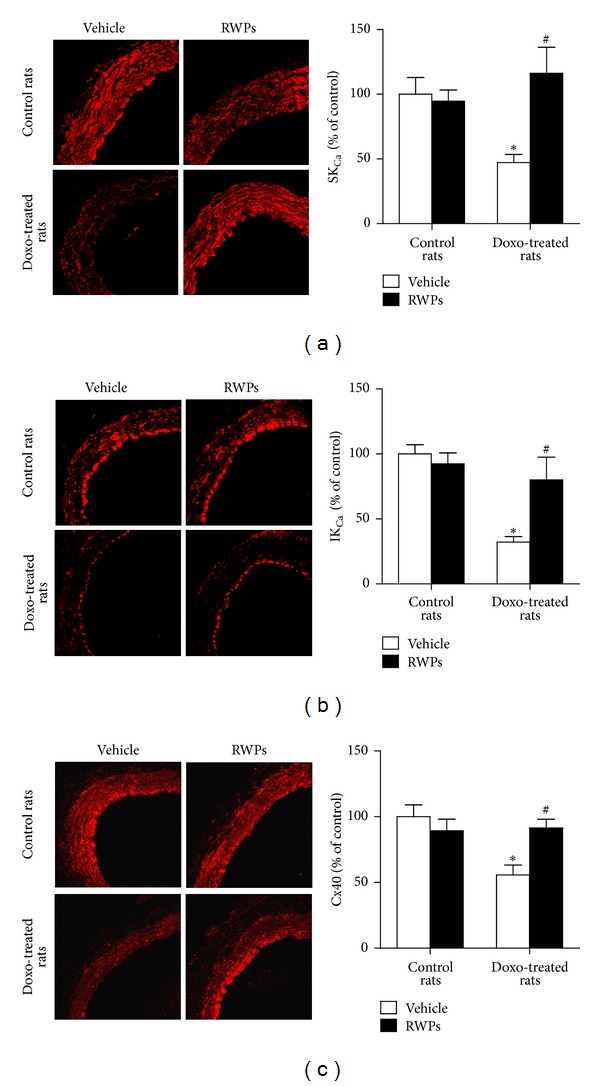
RWPs treatment prevents the doxorubicin-induced impaired expression levels of SK_Ca_, IK_Ca_, and Cx40 in the mesenteric artery. The expression levels of  SK_Ca_, IK_Ca_, and Cx40 were determined using purified polyclonal antibodies and a fluorescence-tagged secondary antibody by confocal microscopy. Left panels show representative immunofluorescent staining, and right panels show corresponding cumulative data. Results are shown as mean ± SEM of 4 different rats. *P < 0.05 indicates a significant effect versus control rats, and ^#^ *P* < 0.05 versus doxorubicin-treated rats.

Since the RWPs treatment improved the doxorubicin-induced blunted EDH-mediated relaxations, the vascular expression levels of SK_Ca_, IK_Ca_, and Cx40, which are all involved in EDH responses, were assessed in the arterial wall by immunofluorescence. The expression levels of SK_Ca_, IK_Ca_, and Cx40 were significantly reduced in the mesenteric artery of doxorubicin-treated rats compared with the control rats, whereas no such effect was observed in rats receiving RWPs plus doxorubicin and also not in those receiving only RWPs (Figure 2).

### 3.3. RWPs Treatment Prevents the Doxorubicin-Induced Vascular Formation of ROS and Upregulation of eNOS Expression in the Mesenteric Artery

**Figure 3:**
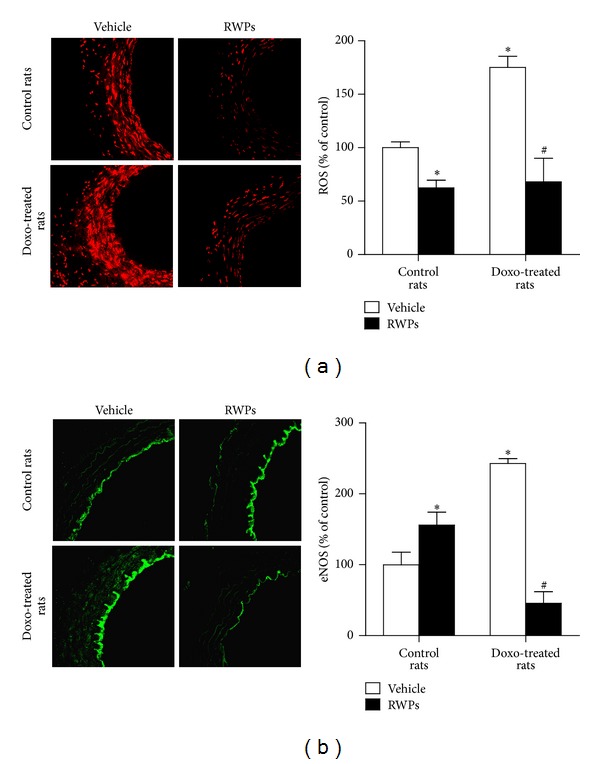
RWPs treatment prevents the doxorubicin-induced vascular oxidative stress and upregulation of eNOS expression in the mesenteric artery. Mesenteric arterial sections were exposed to the redox-sensitive fluorescent dye DHE for 30 min at 37°C. Thereafter, ethidium fluorescence was determined by confocal microscopy. The expression level of eNOS was determined by immunofluorescence. Left panels represent ethidium (a) and immunofluorescent (b) staining, and right panels represent corresponding cumulative data. Results are shown as mean ± SEM of 3 to 4 different rats. *P < 0.05 indicates a significant effect versus control rats, and ^#^ *P* < 0.05 versus doxorubicin-treated rats.

**Figure 4:**
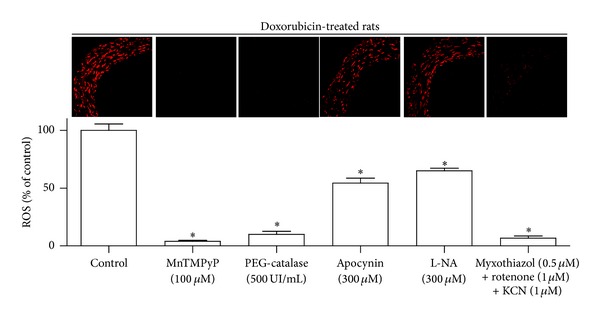
Characterization of the doxorubicin-induced vascular oxidative stress in the mesenteric artery. Mesenteric artery sections from doxorubicin-treated rats were exposed to either MnTMPyP (membrane-permeant superoxide dismutase mimetic), PEG-catalase (membrane-permeant catalase), apocynin (a NADPH oxidase inhibitor and antioxidant), L-NA (NO synthase inhibitor), or inhibitors of the mitochondrial respiration chain (KCN, myxothiazol, and rotenone) for 30 min before DHE staining. Thereafter, ethidium fluorescence was determined by confocal microscopy. Upper panel represents ethidium staining, and lower panel represents corresponding cumulative data. Results are shown as mean ± SEM of 4 different rats. *P < 0.05 indicates a significant effect versus control.

Since doxorubicin has been shown to increase the vascular formation of ROS [4, 5], the possibility that the RWPs treatment improves the doxorubicin-induced vascular oxidative stress was assessed using the redox-sensitive fluorescent probe DHE. The formation of ROS was markedly increased throughout the entire mesenteric arterial wall of doxorubicin-treated rats compared with control rats, whereas no such effect was observed in rats treated with RWPs and doxorubicin (Figure 3(a)). Administration of RWPs alone decreased slightly the vascular DHE fluorescence signal (Figure 3(a)). Immunofluorescence staining of eNOS in mesenteric artery sections indicated an increased endothelial staining in doxorubicin-treated rats compared with control rats, whereas such an effect was prevented by the RWPs treatment (Figure 3(b)). In addition, intake of RWPs alone increased slightly but significantly eNOS staining compared with control rats (Figure 3(b)). In order to determine the nature and source of ROS, arterial sections from doxorubicin-treated rats were treated with different pharmacological tools, and the formation of ROS was examined. The enhanced formation of ROS in the mesenteric arterial wall of doxorubicin-treated rats was markedly reduced by membrane-permeant analogs of either superoxide dismutase (MnTMPyP) or catalase (PEG-catalase; Figure 4). It was also reduced by apocynin (a NADPH oxidase inhibitor and antioxidant), by L-NA (NO synthase inhibitor), and markedly by inhibitors of the mitochondrial respiration chain (KCN, myxothiazol, and rotenone) indicating that the doxorubicin-induced vascular formation of ROS involves an intracellular formation of superoxide anions and hydrogen peroxide, which are generated by NADPH oxidase, uncoupled eNOS, and, in particular, the mitochondrial respiration chain (Figure 4).

### 3.4. RWPs Treatment Prevents the Doxorubicin-Induced Vascular Upregulation of Angiotensin II and AT1 Receptor Expression Levels

**Figure 5:**
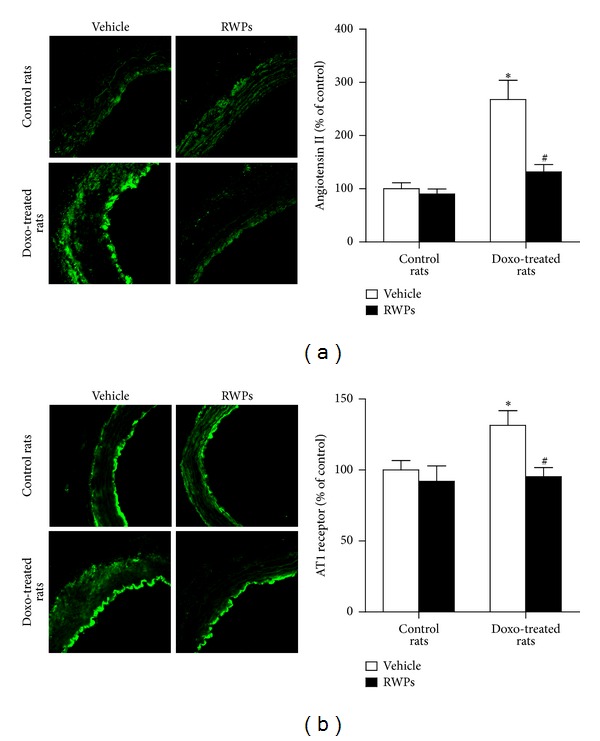
RWPs treatment prevents the doxorubicin-induced expression levels of angiotensin II and AT1 receptors in the mesenteric artery. The expression levels of angiotensin II (Ang II) and AT1 receptors were determined by immunofluorescence. Left panels show representative immunofluorescent staining, and right panels show corresponding cumulative data. Results are shown as mean ± SEM of 4 different rats. *P < 0.05 indicates a significant effect versus control rats, and ^#^ *P* < 0.05 versus doxorubicin-treated rats.

Since activation of the angiotensin system has been implicated in the doxorubicin-induced cardiotoxicity [16], the expression levels of angiotensin II and AT1 receptors were assessed by immunofluorescence staining in the mesenteric artery. The expression levels of angiotensin II and AT1 receptors were significantly increased in the mesenteric artery of doxorubicin-treated rats predominantly at the luminal site (Figure 5). Chronic intake of RWPs prevented the doxorubicin-induced expression levels of angiotensin II and AT1 receptors in the mesenteric artery (Figure 5). In addition, the RWPs treatment alone affected neither the angiotensin II nor the AT1 receptors expression levels (Figure 5).

## 4. Discussion

The present findings indicate that chronic *in vivo* administration of doxorubicin to rats is associated with the induction of an endothelial dysfunction affecting selectively the EDH component of the relaxation, whereas the NO component is little affected in the mesenteric artery. Previous studies have shown that doxorubicin also significantly reduced endothelium-dependent NO-mediated relaxations in large elastic arteries such as the rat and rabbit aortae [4, 14, 25]. This difference may be due to the fact that in large elastic arteries such as aortae NO accounts solely for endothelium-dependent relaxations, whereas in the mesenteric artery both the NO component and EDH component of relaxation are equally important [26]. It may also be explained by differences in the frequency as well as dose of doxorubicin administered that the previous studies used a single high dose of doxorubicin (10 mg/kg for the rabbit and 20 mg/kg for the rat), while the present study used 6 injections of a low dose of doxorubicin (2.5 mg/kg). Moreover, the present study investigated the long-term (6 weeks after the last injection) effects of doxorubicin, whereas previous studies reported an endothelial dysfunction in response to a shorter duration (within 12 h or 7 days) of doxorubicin IP injection. An endothelial dysfunction involving a selective blunted EDH-mediated relaxation in mesenteric artery rings has also been observed in spontaneously hypertensive rats [27] and in angiotensin II-treated rats [28]. In healthy arteries, EDH-mediated relaxations involve activation of SK_Ca_ and IK_Ca_ channels leading to the hyperpolarization of the endothelium, which is then transmitted to the underlying vascular smooth cells, in part, via myoendothelial gap junctions formed by Cx37, Cx40, and Cx43 to cause vasorelaxation [12]. A critical role has been attributed to Cx40 in EDH-mediated relaxations in the rat mesenteric artery since inhibition of Cx40 using antibodies and mimetic peptides markedly depressed EDH-mediated relaxation, whereas inhibition of Cx37 and Cx43 had little effect [29]. The present findings indicate that chronic treatment with doxorubicin reduced the expression levels of IK_Ca_, SK_Ca_, and Cx40, suggesting that the impaired EDH-mediated relaxation is due, at least in part, to a reduced expression of calcium-activated potassium channels and Cx40-dependent gap junctions. Blunted EDH-mediated relaxations in the rat mesenteric artery have also been associated with decreased expression, of SK_Ca_ and IK_Ca_ in angiotensin II-induced hypertension [30], chronic bile duct ligation-induced portal hypertension [31], and middle-aged rats [32], with decreased expression levels of Cx37 and Cx40 in the mesenteric artery of spontaneously hypertensive rats [33], and with a decreased expression levels of Cx37, Cx40, and Cx43 in the mesenteric artery of angiotensin II-treated rats [28]. In addition, the doxorubicin treatment did affect endothelium-independent relaxations neither to a NO donor (sodium nitroprusside) nor to an ATP-sensitive K^+^ channel opener (levcromakalim), indicating that the function of the smooth muscle was not affected by the doxorubicin treatment. In contrast, previous studies have indicated that doxorubicin impaired endothelium-independent relaxations to sodium nitroprusside in the rabbit [14] and rat aortae [4]. Such a difference may be due to the fact that different protocols and doses of doxorubicin have been used. 

The characterization of the mechanism underlying the doxorubicin-induced cardiotoxicity and endothelial dysfunction has indicated the involvement of vascular oxidative stress [3, 5]. The present findings are consistent with those previous ones and indicate an increased oxidative stress throughout the mesenteric artery wall of the doxorubicin-treated rats. They further indicate that the vascular oxidative stress involves both superoxide anions and hydrogen peroxides generated by NADPH oxidase, uncoupled eNOS, and, in particular, the mitochondrial respiration chain. The doxorubicin-induced impairment of the EDH-mediated relaxation is most likely initiated by the increased vascular formation of ROS since oxidative stress has been associated with rapid blunting of EDH-mediated responses [34]. In contrast, NO-mediated relaxations were not affected by the doxorubicin treatment despite an increased oxidative stress and eNOS expression. The increased expression of eNOS is most likely part of a compensatory mechanism since eNOS appears to be partially uncoupled resulting in the generation of superoxide anions rather than NO [35, 36].

Previous studies have indicated that activation of the angiotensin system is involved in the development of the doxorubicin-induced cardiotoxicity [16, 17, 37]. Indeed, doxorubicin failed to induce myofibrillar loss and to increase the number of apoptotic cardiomyocytes in AT1 knockout mice and also in wild-type mice treated with an AT1 receptor antagonist [16]. In addition, the angiotensin-converting enzyme inhibitor, enalapril, attenuated doxorubicin-induced cardiac dysfunction associated with an improvement of the mitochondrial respiratory efficiency and a reduced level of oxidative stress [17]. The AT1 receptor antagonist telmisartan also reduced the doxorubicin-induced expression of the NADPH oxidase subunits p22^phox^, p47^phox^, p67^phox^, and Nox4 and the nuclear factor kappa B in the heart [38]. In addition, both telmisartan and captopril induced similar protective effects against doxorubicin-induced cardiotoxicity and nephrotoxicity, in part, by reducing oxidative stress [37]. In agreement with those previous observations, the present findings also support a key role for the angiotensin system in the doxorubicin-induced endothelial dysfunction since increased expression levels of both angiotensin II and AT1 receptors were observed in the arterial wall of doxorubicin-treated rats.

The chronic treatment of doxorubicin-treated rats with RWPs (75 mg/kg/day in the drinking water) prevented the doxorubicin-induced blunted EDH-mediated relaxations in the rat mesenteric artery. The beneficial effect is associated with improved vascular expression levels of SK_Ca_ and IK_Ca_ channels and Cx40 possibly due to the fact that the RWPs treatment prevented the doxorubicin-induced vascular oxidative stress [34]. Moreover, the RWPs treatment normalized the expression levels of angiotensin II and AT1 receptors in the mesenteric artery of doxorubicin-treated rats to levels similar to those observed in control rats. This effect is most likely also due to the antioxidant effect of RWPs since oxidative stress has been associated with the activation of the local renin-angiotensin system in the uric acid-induced endothelial dysfunction [39], and also the upregulation of AT1 receptors in the rat kidney [40, 41]. The ability of polyphenols to inhibit the angiotensin-converting enzyme might contribute to the decrease of expression of angiotensin II in doxorubicin-treated rats receiving RWPs [42]. 

In conclusion, doxorubicin-induced an endothelial dysfunction in the rat mesenteric artery, which is characterized by blunted EDH-mediated relaxations associated with reduced vascular expression levels of SK_Ca_, IK_Ca_, and Cx40 and vascular oxidative stress. The RWPs treatment prevented the doxorubicin-induced endothelial dysfunction most likely by improving the excessive vascular formation of ROS and the activation of the angiotensin system.
